# Treatment of HIV among tuberculosis patients: A replication study of timing of antiretroviral therapy for HIV-1-associated tuberculosis

**DOI:** 10.1371/journal.pone.0210327

**Published:** 2019-02-01

**Authors:** Eric W. Djimeu, Anna C. Heard

**Affiliations:** 1 International Initiative for Impact Evaluation (3ie), Washington, DC, United States of America; 2 CEREG, University of Yaoundé II, Yaoundé, Cameroon; Management Sciences for Health, ETHIOPIA

## Abstract

Co-diagnosis of HIV and tuberculosis presents a treatment dilemma. Starting both treatments at the same time can cause a flood of immune response called immune reconstitution inflammatory syndrome (IRIS) which can be lethal. But, how long to delay HIV treatment is less understood. In 2011, based on the conclusions of three separate studies, WHO recommended starting HIV treatment earlier for those with later HIV disease progression. This paper conducts a replication study of one of the three studies, by Havlir and colleagues. Using their publicly available data, we were able to replicate most of the results presented in the original paper. In our measurement and estimation analyses we use different estimation techniques to assess the robustness of the results. We find that adjusting for loss to follow-up does not affect the main results of the paper. However, an ANCOVA estimation and an instrumental variable model weaken the main result of the paper of better outcomes with early HIV treatment only for those who are sicker, reducing significance from the 5% to the 10% level. A change-point analysis also detects no changes in effect by timing of HIV treatment initiation or different thresholds of CD4 count for the primary outcome. This result suggests that the choice of start time for HIV treatment initiation should be based on other factors including potential drug interactions, overlapping side effects, a high pill burden and severity of illness rather than CD4 threshold and preset timeframes. While we caution against overgeneralizing, the result of this replication is aligned with more recent studies that show no evidence that early initiation of HIV treatment reduces mortality for any patients.

## 1. Introduction

According to the World Health Organization (WHO), Tuberculosis (TB) is the most common presenting illness and leading cause of death among people living with HIV (PLWHIV) [1]. TB-HIV co-infection is so common that routine HIV testing is recommended for all TB patients and isoniazid preventive therapy is recommended for all PLHIV [1]. In 2011, when seminal papers were published on the timing of TB and HIV co-treatment, an estimated 1.1 million people globally were newly co-diagnosed with HIV and TB, and 79% of TB-HIV co-infected patients lived in sub-Saharan Africa [1].

For TB, the standard treatment starts with an eight week intensive phase followed by four months of continuation therapy. In co-infected patients, TB is treated first. WHO-recommended HIV treatment eligibility has changed over time. Due to high mortality associated with TB for HIV patients, WHO has recommended universal access to antiretroviral therapy (ART) for HIV-positive TB patients irrespective of CD4 count [2]. Prior research suggested that co-treatment is safe [2], but concerns about an immune response, immune reconstitution inflammatory syndrome (IRIS) and potential drug interactions and side effects demanded more evidence on when to start ART. Three studies were conducted to determine the optimal timing for ART initiation [3, 4, 5].

Havlir et al., used for this replication, was a large multisite trial conducted in 26 countries. It found that earlier ART (within 2 weeks of TB treatment initiation) reduced the rate of new AIDS defining illness or death (hereafter AIDS event) exclusively in persons with CD4 counts of less than 50 as compared with later ART (between 8 and 12 weeks after TB treatment initiation) [6]. The study conducted in South Africa found similar results, although the effect on mortality was not significant (IRR=0.32, p=0.06) and defined early as within 4 weeks of starting TB treatment [3]. The study conducted in Cambodia, used a CD4 count cut-off of 200, and found that earlier treatment (2 weeks after beginning TB treatment) reduced the risk of death in patients with lower CD4 counts compared with later ART (8 weeks after) [5]. However, the study population was generally more sick (median CD4 = 25 as compared to 150 for [4] and 77 for [5]).

Based on these three studies, in 2011 WHO recommended (and still recommends) beginning ART within eight weeks of initiation of TB treatment in co-infected patients with a CD4 count higher than 50 and within two weeks for those with a CD4 count lower than 50. Replication of influential studies, studies that are the basis of a major policy or program change or decision, is important to provide added confidence that the findings are real and robust. This is especially true when the policies are based on only one or a few studies or a limited amount of evidence.

We selected Havlir et al. for replication for three main reasons. First, the three studies continue to be influential. The WHO guidelines still recommend earlier initiation of ART in co-infected patients with a CD4 count under 50. Second, Havlir et al. uses the same parameters as the WHO recommendations (within 2 weeks and CD4 below 50), includes more observations than the other three studies and was conducted in four continents, potentially providing greater statistical power, enabling greater sub-group analysis and greater external validity. This replication will confirm its robustness. Third, the lack of definitive evidence for earlier versus delayed ART for persons with CD4 counts of 50 or more is highlighted in a recent systematic review on the timing of ART initiation [7]. In particular, while their meta-analysis strongly supports early ART initiation in adults with CD4 counts of less than 50, they found insufficient evidence to draw conclusions about the timing of ART initiation for patients when CD4 counts are between 50 and 220. The analysis also identified an increased risk of IRIS with early treatment, and suggested it may be safe to delay ART initiation until after full TB treatment is completed in patients with CD4 counts over 220. The authors called for more evidence and suggested that the WHO update their guidelines.

More recently four other studies have been conducted on the timing of ART initiation and present evidence contrary to those initial findings. All fail to find a difference in mortality/survival rates between earlier and later ART initiation. A study conducted in Thailand of patients with CD4 counts <350 (median baseline CD4 = 43) and active TB found no advantage of early treatment (at 4 weeks) compared with ART initiation at 12 weeks for any measure, including for patients with CD4<50 or CD4 <100 [8]. In a similar study conducted in India, the authors found no difference in mortality between 88 patients who initiated ART after 2-4 weeks and 62 patients who initiated ART within 8-12 weeks, including no difference for patients with a baseline CD4<100. Median baseline CD4 count was 133 [9]. In a study conducted at 26 centers in South Africa, Tanzania, Uganda and Zambia that enrolled HIV-positive patients with CD4 counts of 220 or more, the authors find that mortality did not differ significantly between earlier ART and delayed ART [10]. Finally, a study conducted in Ethiopia randomized the initiation of ART to one week, four weeks, and eight weeks in patients with a baseline CD4 count of < 200 (median CD4 = 73), found that ART at one week after TB treatment didn’t improve overall survival compared to 4 and 8 weeks. Although the delayed start for those with CD < 50 may slightly increase their mortality rates, the authors recommend initiation of ART later than the first week of TB treatment, regardless of CD4 count, to avoid serious hepatotoxicity and treatment interruption [11].

Overall, we find the evidence mixed as to whether earlier initiation of ART reduces mortality, even in patients with lower CD4 counts. More recent studies fail to find differences, while a systematic review that includes the earlier studies [7] found that for these patients there was an advantage. In addition, there remain questions about patients with CD4 counts between 50 and 200, and the definition of “earlier” and “later” initiation is not uniform across studies and the rationale for the selection or definition of earlier and later is not always explained in these studies. It is clear that the evidence regarding the optimal timing of ART initiation requires careful review so that one can verify and understand better the results of the three studies used by WHO in their treatment guidelines [2].

Thus, this replication study will contribute to filling the knowledge gap regarding the uncertainty around delaying ART for HIV-TB co-infected patients with CD4 counts between 50 and 220. Neither the CD4 cut-off point nor the timing definition of “early” and “later” seems well-justified in any of the three studies. Differences between studies suggest that the selection is somewhat arbitrary. This replication will, therefore, use data from the selected study to assess whether there is an endogenously defined cut-off outside of which the effect of ART on mortality changes.

The remaining sections of this paper are organized as follows. Section 2 presents the pure replication. Section 3 presents the measurement and estimation analysis (MEA) and section 4 concludes.

## 2. Pure replication

A pure replication consists of re-conducting the original analyses using the data and statistical methods of the original paper [12]. In the next sections, we describe the data used for this replication, statistical methods of the original paper, and pure replication results.

### 2.1 Study population of the original paper and data used for the replication

Participants in the original study were from 26 countries in Africa, Asia, North America and South America. Eligible participants were 13 years of age or older, had HIV-1 infection with a CD4 count of less than 250 (the CD4 count threshold for HIV treatment eligibility at the time), had not previously received ART, and had confirmed or probable TB. From September 2006 through August 2009, 809 patients were enrolled in the study.

For this replication study we use the SAS data file A5202ANIN_2011.trn, prepared by the Center for Biostatistics in AIDS Research, Harvard School of Public Health and distributed by National Technical Information Service. This dataset includes all information collected during the trial and used for the original study aside from a few indicators. The public data were de-identified—key variables that could potentially be used to identify an individual were removed or modified: Age was grouped, all dates were converted to the number of days, race, continent, site and institution information were removed. The participant identifier is a random number and not the original identifier.

We constructed all variables required for the replication by using the raw, publicly available data. The data did not include the statistical code used to produce results presented in the original paper and in some cases there was insufficient information to construct some variables used in the original paper.

We were thus required to contact the original authors directly to get clarification on how to construct them. We closely followed their instructions and do not anticipate that the construction of these variables, nor the information removed to de-identify the original data, will affect the comparability of the results of the pure replication with the main results in the original paper.

We converted the SAS data to Stata format and used Stata 14.1 to conduct the pure replication study.

### 2.2 Statistical methods used in the original paper

Original authors used the Kaplan-Meier method to assess the impact of earlier ART initiation on the primary endpoint (AIDS event), a standard epidemiological model for a time-to-event outcome. Kaplan-Meier is a nonparametric method to calculate the cumulative survival over time, taking into account differing risk sets at each time point related to individuals lost to follow-up, still at risk, or having already experienced the outcome [13]. The original authors also used a Pearson chi-square test to compare rates of a new AIDS event at 48 weeks (primary endpoint). They estimated proportions of patients who survived without an event at endpoint. Tests were stratified by sub-group. Three sub-group analyses were pre-specified [5], CD4 count strata (<50 and ≥50), level of TB diagnostic certainty (probable or confirmed) and body mass index (BMI) (≤18.5 and >18.5), to assess for heterogeneous treatment effects.

The authors also assessed between-group differences in baseline characteristics of the patients and secondary endpoints. The secondary endpoints included HIV viral load and immune response to ART (change in CD4 count) at 48 weeks, adverse events attributed to TB associated IRIS at 48 weeks, and other serious adverse events at 48 weeks (Grade 3 or 4 Clinical Events or Laboratory Abnormalities).

We replicated all the tables and figures using the same methods used in the original paper.

### 2.3 Pure replication results

We were able to replicate all the baseline characteristics of patients, barring those removed from the public databases (country and age at enrollment) (Table 1). Except for this, our baseline results are identical to those presented in the original paper. In addition, we confirm in all but two cases that baseline characteristics are balanced between the two study arms. The baseline interval (days) between start of TB therapy and start of ART is set for study participants and thus different, consistent with the design of the study and with the original results. However, while we are able to confirm the same number and proportion of male participants in each arm (266 and 66% in the earlier versus 235 and 59% in the later ART), we find that the proportions are significantly different at 5% (p=0.04), which was not noted by the original authors. We classify this as a major difference.

**Table 1:** Baseline characteristics of the patients.

	Panel A: Original results			Panel B: Replication results		
Characteristics	Earlier ART (N=405)	Later ART (N=401)	All patients	Earlier ART (N=405)	Later ART (N=401)	All patients
Continent – no. (%)						
Africa	275 (68)	279 (70)	554 (69)	N/A	N/A	N/A
Asia	29 (7)	23 (6)	52 (5)	N/A	N/A	N/A
North America	21 (5)	18 (4)	39 (5)	N/A	N/A	N/A
South America	80 (20)	81 (20)	161 (20)	N/A	N/A	N/A
Male sex – no. (%)	266 (66)	235 (59)	501 (62)	266 (66)	235 (59)	501 (62)
Age at enrollment – yr						
Median	34	34	34	N/A	N/A	N/A
Interquartile range	29-40	29-42	29-41	N/A	N/A	N/A
TB – no. (%)						
Confirmed	193 (48)	181 (45)	374 (46)	193 (48)	181 (45)	374 (46)
Probable	208 (51)	218 (54)	426 (53)	208 (51)	218 (54)	426 (52)
Not TB	4 (1)	2 (<1)	6 (1)	4 (1)	2 (<1)	6 (1)
CD4+ T-cell						
Median	70	82	77	70	82	77
Interquartile range	34-146	40-144	36-145	34-146	40-144	36-145
HIV-1 RNA						
Median	5.39	5.5	5.43	5.39	5.49	5.42
Interquartile range	4.94-5.79	5.03-5.79	5.00-5.79	4.39-5.79	5.03-5.79	5.00-5.79
Prior AIDS	26 (6)	29 (7)	55 (7)	26 (6)	29 (7)	55 (7)
Body-mass index						
Median	19.1	19.4	19.2	19.0	19.4	19.2
Interquartile range	17.3-21.1	17.7-21.8	17.5-21.4	17.3-21.1	17.7-21.8	17.5-21.4
Initial ART regimen of EVF/FTC/TDF* – no./total no. (%)	394/403 (98)	368/380 (97)	762/783 (97)	392/403(97)	368/380(97)	760/783(97)
Interval TB_ART						
Median	10	70		10	70	
Interquartile range	7-12	66-75		7-12	66-75	

*EVF/FTC/TDF also known as Atripla: efavirenz, emtricitabine, tenofovir disoproxil fumarate

Notes: We shade results from this replication study to indicate discrepancies we detected between the original results and results from the reanalysis. In this table, we were not able to replicate the results due to missing variables. This is not a comment on the original author’s methods or on the quality of their work. Panel A Source: Author’s’ construction using the SAS data file A5202ANIN_2011.trn, prepared by Center for Biostatistics in AIDS Research Harvard School of Public Health and distributed by National Technical Information Service

To the best of our knowledge, there is no standard definition or rule of thumb for classifying major or minor differences in replication studies. Here, we classify a difference as major when the significance level of a coefficient changes or when the difference in effect size between the original results and the replication results is larger than 10%.

Table 2 presents rates of new AIDS events at 48 weeks, according to CD4 count. In general, although the numbers of patients in panel A (original paper results) and panel B (replication results) are identical, the proportions in panel A are slightly larger than the proportion in panel B. We are unable to determine why the proportion with AIDS events appears lower with the data we have. The total number of AIDS events appear the same in the original paper and in our replication results. Except in one sub-group, p-values from the pure replication are generally similar to those presented in the original paper and importantly these p-values have the same level of significance. The exception is for patients with low BMI and CD4 count <50 at study entry. The difference in outcome in the earlier ART arm and the later ART arm is statistically significant at 5% whereas the original authors reported it as significant at 1%. In short, for most of the indicators, there are only small or no differences.

**Table 2:** Rates of new AIDS-defining illness or death at 48 weeks, by CD4+ count.

	Panel A: Original results					Panel B: Replication results				
Variables	N	Earlier ART (%)	Later ART (%)	95% CI	P-Value	N	Earlier ART (%)	Later ART (%)	95% CI of diff in mean	P-Value
All Patients	806	12.9	16.1	-1.8 to 8.1	0.45	806	12.8	16	-1.7 to 8.0	0.21
<50 cells/mm^3^	285	15.5	26.6	1.5 to 20.0	0.02	285	15.3	26.2	1.6 to 20.4	0.02
≥50 cells/mm^3^	521	11.5	10.3	-6.7 to 4.3	0.67	521	11.5	10.4	-6.5 to 4.3	0.68
Confirmed TB at study entry	374	13.8	19.7	-1.8 to 13.6	0.21	374	11.9	17.1	-1.9 to 12.4	0.15
<50 cells/mm^3^	151	17.9	31.4	-0.4 to 27.3	0.06	151	16.3	28.2	-1.3 to 25.1	0.08
≥50 cells/mm^3^	223	10.8	12.1	-7.3 to 9.8	0.77	223	8.9	10	-6.6 to 8.9	0.77
Suspected TB at study entry	432	15.4	19.7	-3.0 to 11.7	0.35	432	13.7	15	-5.3 to 8.0	0.70
<50 cells/mm^3^	134	14.1	30.5	2.5 to 30.4	0.02	134	14.1	24.3	-3.3 to 23.7	0.14
≥50 cells/mm^3^	298	15.9	14.5	-9.8 to 7.1	0.75	298	13.5	10.7	-10.2 to 4.6	0.45
Low BMI (≤18.5) at study entry	332	16.3	26.5	1.2 to 19.2	0.06	332	14.9	23.2	-1.6 to 16.7	0.05
<50 cells/mm^3^	130	15.2	38.2	8.0 to 37.8	0.003	130	15.2	32.8	3.1 to 32.2	0.02
≥50 cells/mm^3^	202	16.9	17.8	-9.9 to 11.6	0.88	202	14.8	16.1	-8.8 to 11.5	0.80

Notes: We shade results from this replication study to indicate discrepancies we detected between the original results and results from the reanalysis. Panel A Source: Author’s’ construction using the SAS data file A5202ANIN_2011.trn, prepared by Center for Biostatistics in AIDS Research Harvard School of Public Health and distributed by National Technical Information Service. CD4 levels were “stratified” for lower (<50) and higher (>=50), but some “sick” patients were classified as low-BMI

**Fig 1:**
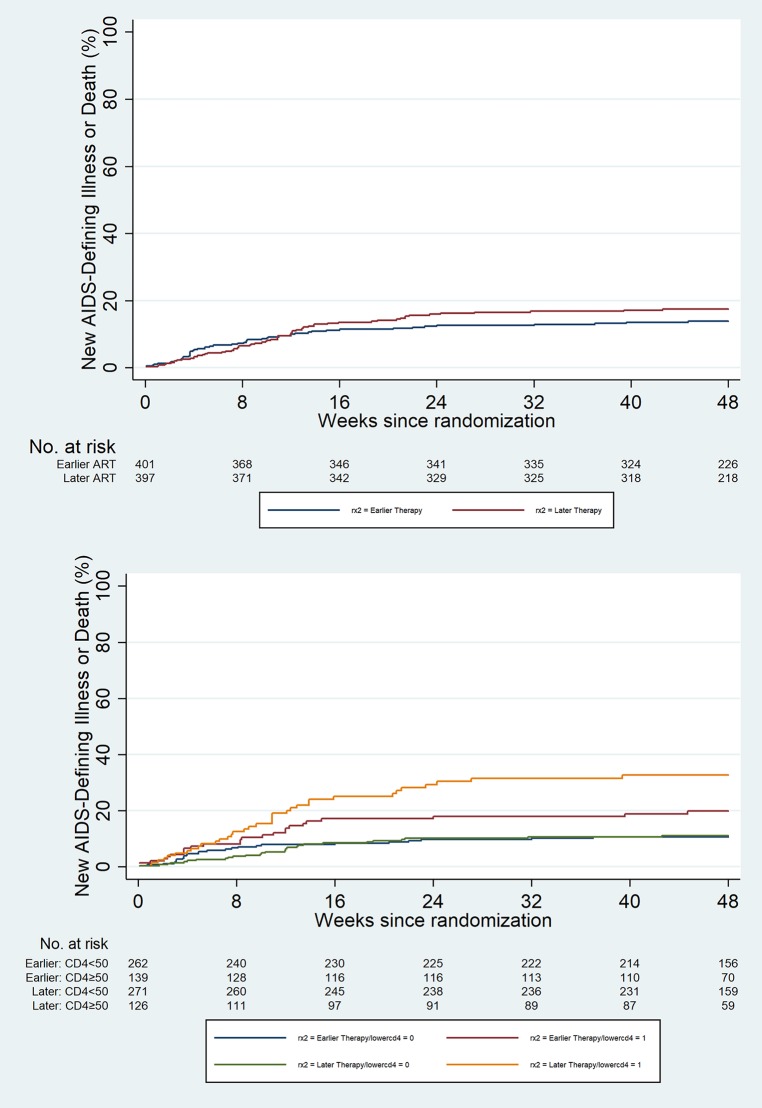
Time to new AIDS-defining illness or death—replicated Kaplan-Meier curves. Note: The original results can be found in Fig 2 in [5], which is open access.

Fig 1 presents the replicated Kaplan-Meier unadjusted survival curves between the two study arms. The original paper curves could not e printed here for copyright reasons. They can be found in [5].

Table 3 presents results of HIV RNA level and immune response to antiretroviral therapy. The replication results are similar to original paper. However, the definition used for viral suppression (or “undetectable” RNA) in the original paper was <400 copies/ml, but to replicate the results we needed to use a definition of ≤400. This is likely due to the use of two different tests for viral load, one more sensitive than the other with the less sensitive reporting the lowest level as “present, <400 copies/ml” and the other able to detect as low as 50 copies per ml. Examination of viral load level distribution indicated heaping at 400 and 50, suggesting that the lowest levels were converted to 400 and 50 for convenience. For accurate replication the original authors should have defined viral suppression at ≤400, rather than the often standard definition of <400.

**Table 3:** HIV RNA level and immune response to ART.

	Panel A: Original results					Panel B: replication results				
Outcome	WK 8	WK 16	WK 24	WK 32	WK 48	WK 8	WK 16	WK 24	WK 32	WK 48
HIV-1 RNA <400 copies/ml — no./total no. (%)										
Earlier ART	273/370 (74)	314/361 (87)	320/355 (90)	313/349 (89)	293/331 (89)	275/370 (74)	316/361 (87)	322/355 (90)	316/349 (90)	296/331 (89)
Later ART	4/380 (1)	237/365 (65)	295/349 (85)	313/347 (90)	301/332 (91)	5/380 (1)	240/365 (65)	298/349 (85)	313/347 (90)	301/333 (90)
CD4+ T-cell count										
Earlier ART										
No. of patients	368	357	350	346	333	368	357	350	346	333
Median –cells	200	207	218	219	246	195	207	217.5	218.5	250
Interquartile range	121 to 275	134 to 279	145 to 294	154 to 305	169 to 352	117 to 271	136 to 280	143 to 293	154 to 305	169 to 354
Later ART										
No. of patients	379	364	347	343	333	379	364	347	343	333
Median –cells	77	193	207	221	250	78.5	183	205	221	250
Interquartile range	34 to 140	113 to 289	130 to 296	150 to 308	173 to 343	36 to 143	104 to 279	129 to 293	149 to 306	177 to 339
Change from baseline in CD4+ T-cell count										
Earlier ART										
No. of patients	368	357	350	346	333	368	357	350	346	333
Median –cells	93	107	124	132	160	91	107	122.5	132	160
Interquartile range	48 to 172	5 to 187	72 to 189	77 to 198	91 to 240	57 to 171	65 to 167	72 to 187	77 to 198	90 to 240
Later ART										
No. of patients	379	364	347	343	333	379	364	347	343	333
Median –cells	-2	95	104	124	151	-2	85.5	103.5	124	151
Interquartile range	-23 to 14	43 to 165	60 to 173	71 to 204	94 to 228	-23 to 17	34 to 162.5	59 to 172.5	71 to 202	94 to 228

Notes: We shade results from the replication study to indicate discrepancies we detected between the original results and results from the reanalysis. Panel A Source: Author’s construction using the SAS data file A5202ANIN_2011.trn, prepared by Center for Biostatistics in AIDS Research Harvard School of Public Health and distributed by National Technical Information Service.

Finally, Table 4 presents results for severe (grade 3) or life threatening events (grade 4) and laboratory abnormalities. We find two minor differences. Our replication results show a lower proportion of patients with neurologic events and a higher proportion of patients with any severe or life threatening adverse event compared to the original result. There is not sufficient information to explain these differences, although it is likely due to differences in how events were categorized. Despite several exchanges with the statistician who did the analysis, we were unable to replicate the results presented in the original paper.

**Table 4:** Grade 3 or 4 clinical events or laboratory abnormalities.

	Panel A: Original results			Panel B: Replication results		
Event	Earlier ART (N=405)	Later ART (N=401)	Total (N=806)	Earlier ART (N=405)	Later ART (N=401)	Total (N=806)
	Number of patients (percent)			Number of patients (percent)		
Clinical event						
Constitutional	31 (8)	31 (8)	62 (8)	33 (8)	32 (8)	65 (8)
Respiratory	17 (4)	16 (4)	33 (4)	21 (5)	15 (3)	36 (4)
Cardiac or Circulatory	11 (3)	7 (2)	18 (2)	10 (2)	4 (<1)	14 (1)
Gastrointestinal	17 (4)	20 (5)	37 (5)	17 (4)	20 (5)	37 (4)
Cutaneous	11 (3)	11 (3)	22 (3)	8 (1)	10 (2)	18 (2)
Neurologic	22 (5)	28 (7)	50 (6)	8 (1)	4 (<1)	12 (1)
Laboratory abnormality						
Absolute neutrophil count<750/mm^3^	36 (9)	69 (17)	105 (13)	41 (10)	92 (22)	133 (16)
Hemoglobin<7.5g/dl	28 (7)	22 (5)	50 (6)	46 (11)	30 (7)	76 (9)
Platelet count<50,000/mm^3^	3 (1)	13 (3)	16 (2)	2 (1)	16 (3)	18 (2)
Aminotransferase>5x ULN	26 (6)	41 (10)	67 (8)	30 (7)	43 (10)	73 (9)
Creatinine>1.9x ULN	12 (3)	7 (2)	19 (2)	13 (3)	9 (2)	22 (3)
Any laboratory abnormality	65 (16)	55 (14)	120 (15)	67 (16)	55 (13)	122 (15)
Any grade 3 or 4 adverse event	177 (44)	190 (47)	367 (46)	215 (53)	279 (69)	494 (61)

Notes: We shade results from the replication study to indicate discrepancies we detected between the original results and results from the reanalysis. Panel A Source: Author’s construction using the SAS data file A5202ANIN_2011.trn, prepared by Center for Biostatistics in AIDS Research Harvard School of Public Health and distributed by National Technical Information Service

### 2.4 Pure replication conclusions

Using methods from the original analysis we were able to replicate the findings except for two minor and two major differences. We found minor differences of a lower proportion of patients with neurologic events and a higher proportion of patients with any severe or life threatening adverse event compared to the original results. The first major difference is that the difference in proportion of male participants between earlier and later ART initiation groups was not reported as statistically significant, but our replication indicates the same difference is significant at the 5% level. The difference is important because some studies have reported sex differences in HIV outcomes such as virologic failure and viral suppression (see [14,15]) and the difference in proportion of men might thus confound the results. The second major difference, and perhaps most important, is that the replication does not find the difference between earlier and late ART initiation for HIV patients with suspected TB and a CD4 count of less than 50 statistically significant. The original results reported the difference was statistically significant at 5%. This appears to be due to our finding of generally lower percentages of participants with the primary outcome of an AIDS event, especially within the later ART group. This variable was provided in the original dataset and we did not create it, and we find the same total number of AIDS events, so we are unable to determine the cause of this discrepancy.

## 3. Measurement and estimation analysis (MEA)

Our pre-analysis plan, posted on the 3ie website (https://tinyurl.com/yauenyto), states we will assess heterogeneous effects by continent. Due to de-identification (see section on study population and data), these variables were removed and we are unable to perform this assessment.

Although the original authors conducted a thorough analysis, there are still some robustness checks that can be performed. While the Kaplan-Meier method accounts for loss to follow-up through censoring, the Pearson chi-square test of differences at 48 weeks does not. A large and/or differential rate of attrition (loss to follow up) between the earlier and later ART groups could bias the estimation of the effect. We thus assess whether there was differential attrition.

Further, the original study was powered at 80% (a two-sided alpha level of 0.05) to detect a 40% reduction in the rate of treatment failure with later ART versus earlier ART (from 25% to 15%) at 48 weeks. However, many subgroup analyses were conducted implying smaller sample sizes that could result in the assessment being underpowered. For example, one of the main analyses is conducted for patients with CD4 < 50. There are 266 patients with a CD4 < 50. With this sample size the study can only detect a 64% reduction in the rate of treatment failure (25% to 9%). To overcome this potential lack of statistical power, especially for subgroup analysis, we use an ANCOVA estimation, adjusting for baseline CD4 count. Additionally, even though the treatment assignment was random, we found that the difference in proportion of men between groups was significant. We therefore believe that an estimation that adjusts for baseline CD4 values, as well as sex, could be a more precise measure of the effect of earlier treatment. We also use this model to assess differential impact by CD4 group, TB classification, and low BMI.

If the uptake of the intervention (earlier ART) is low, estimates from intention to treat, as was done in the original paper, will provide a lower-bound estimate of the impact of the treatment. We use an instrumental variables approach to estimate the treatment effect on the treated. Unlike restricting the analysis to those who received the treatment, this approach takes into account potential biases related to self-selection that are also correlated with the outcome.

The original paper assessed whether individuals with a CD4 count below 50 responded differently to earlier versus later ART initiation. But the definition for earlier (within 2 weeks) and later (within 8-12 weeks) is somewhat arbitrary—two of the three studies on which WHO based their recommendation used 2 weeks [4, 5], and one used ‘within 4 weeks’ [6]. In fact, there is no evidence that shows two weeks is the appropriate definition for “earlier” as opposed to, for instance, one week or 4 weeks. We propose an endogenous estimation and more systematic exploration of different windows of earlier ART initiation and different cut-off points of CD4 count using change-point analysis. Change point analysis detects subtle changes that are not possible to see in simple trend line plots [16].

In the following sections, we describe our methods and present our MEA results.

### 3.1 Statistical methods used in the MEA

#### 3.1.1 Attrition

Although attrition was low (<10%), we perform a robustness check by comparing attrition rates between the groups using a Pearson chi-square test, and comparing observable characteristics at baseline between those that are lost to follow-up (attritors) and those that remain in the study or have had a qualifying event by 48 weeks using Fisher’s exact or Pearson chi-square for proportions, and Student’s t-test for means.

As an additional robustness check we used regression models to assess whether treatment predicts attrition and to assess whether there were differential effects by different sub-groups. The model uses a treatment indicator, an indicator for being a non-attritor, and an interaction of the two indicators, which indicates whether there is differential selective attrition between participants in the two groups for each sub-group. As a last check on the effect of attrition we restrict the analysis to patients who were not lost to the follow-up (reducing the denominator) and compare the results to the full sample.

#### 3.1.2 Controlling for baseline CD4 and male sex

David McKenzie [17] suggested that an ANCOVA estimation (regression controlling for baseline values) could increase efficiency over a straight difference-in-difference estimator and that more efficiency is gained the lower the autocorrelation. In principle, the lagged outcome variable in this case should be new AIDS events at baseline. However, these two variables are not observed at baseline. For HIV+ people, the CD4 count can be strong predictor of HIV progression [18, 19]. We use the level of CD4 count at baseline as a proxy of the endpoint. The correlation between baseline and an AIDS event at 48 weeks is 0.13, which is relatively low.

In order to assess heterogeneity of effect by CD4 count level, we use the interaction between the baseline CD4 count stratum and treatment group in an ANCOVA estimation using linear and logistic regression. In addition, since sex is imbalanced at baseline, we add a control for male sex.

#### 3.1.3 Treatment of the treated

An IV approach consists of two stages. In the first stage, an instrument is used to predict the compliance to earlier ART initiation status, the treatment. In the second stage, the predicted value of the compliance (rather than treatment assignment) is used to predict the primary endpoint. Random assignment to the treatment group (earlier ART initiation) is a valid instrument for compliance to earlier ART initiation because the probability of starting ART earlier is strongly correlated with the random assignment, and it is related to the outcome of an AIDS event exclusively through earlier ART initiation. Because the IV approach will only result in substantial differences from the intention to treat model if many people are not compliant, we first estimate the compliance, the probability of receiving earlier ART initiation.

#### 3.1.4 Change point analysis

Finally, we use change point analysis to identify the CD4 cut-off point from which earlier ART has no impact on mortality. We use the same approach to determine the optimal start date of earlier ART initiation. In the change point analysis, the critical change point is the point where a major shift in the trend is recognized. This method has been used to detect changes of demographic transition in India [20]. For this analysis we used Change-Point Analyzer downloadable from http://www.variation.com/cpa/index.html.

The output is a figure that displays the trend over time or different levels with lines designating control limits outside which points indicate changes in the trend. The tool provides confidence intervals and estimates for when the change occurred. A detailed description of the method of change point analysis can be found in Taylor [16].

### 3.2 MEA results

#### 3.2.1 Adjusting for loss to follow- up in the analysis

The level of attrition in the earlier ART group is 9.13% and in the later ART group 6.48%. Table 5 presents results for testing differential rates of attrition between groups using linear regression and probit regression. In column 1, we regress a dummy for attrition on an indicator for earlier treatment and, in column 2, we also include array of individual controls. In neither case does the treatment indicator significantly predict attrition. In columns 3 and 4, we perform the same analysis using a probit model to account for the binary outcome of attrition, but the results remain unchanged. Therefore, we conclude that there is no differential attrition between the earlier ART group and the later ART group. In addition, as the attrition rate is generally low, no further efforts, such as imputation methods or Heckman sample selection, were used to adjust for attrition, as was proposed in the replication plan. However, men were more likely to drop out, and due to the imbalance at baseline, estimations should adjust for being male.

**Table 5:** Testing for differential rate of attrition.

	(1) Coeff (SE)	(2) Coeff (SE)	(3) Coeff (SE)	(4) Coeff (SE)
Early ART	0.0265	0.024	0.183	0.169
	(0.02)	(0.02)	(0.13)	(0.13)
Male sex		0.039**		0.292**
		(0.02)		(0.15)
Age		0.024		0.185
		(0.02)		(0.19)
Baseline CD4		0		0.000867
		0.00		(0.00)
Baseline HIV RNA		-0.011		-0.0838
		(0.01)		(0.09)
Baseline BMI		0		-0.00387
		(0.00)		(0.02)
Constant	0.064 ***	0.157	-1.515***	-1.432**
	(0.01)	(0.10)	(0.10)	(0.70)
Observations	806	802	806	802

Notes: Columns 1 and 2, use linear regression; columns 3 and 4, use a probit model. Standard errors in parentheses

*** p<0.01

** p<0.05

* p<0.1

Table 6 reports the results of differences in observables between attritors and non-attritors across treatment groups using the linear regression. We use the linear regression because these regressions are used only to compare attrition rate between the treatment group and the control group along observable characteristics. These regressions are not intended to properly estimate attrition with the best model that fits the data. Interaction coefficients are generally small and none are statistically different from zero. This strongly suggests a lack of selective attrition.

**Table 6:** Differences in observables between attritors and non-attritors across treatment groups.

	(1)	(2)	(3)	(4)	(5)	(6)	(7)
Variables	Age (SE)	Male sex (SE)	CD4 (SE)	HIV RNA (SE)	BMI (SE)	TBconfirmed (SE)	TBsuspected (SE)
Attrition	-0.041	-0.196**	7.526	0.038	-0.521	-0.030	0.030
	(0.077)	(0.098)	(15.840)	(0.139)	(0.709)	(0.101)	(0.101)
Treatment	0.015	-0.076**	(0.653)	(0.024)	-0.636**	0.030	(0.030)
	(0.028)	(0.036)	(5.731)	(0.050)	(0.256)	(0.037)	(0.037)
Non-attrit x treat	0.161	0.116	7.595	-0.201	0.601	-0.0480	0.0480
	(0.102)	(0.129)	(20.790)	(0.181)	(0.930)	(0.133)	(0.133)
Constant	0.811***	1.427***	96.400***	5.353***	20.14***	0.453***	0.547***
	(0.020)	(0.025)	(4.034)	(0.035)	(0.180)	(0.026)	(0.026
Observations	806	806	806	802	806	806	806
R-squared	0.006	0.011	0.002	0.003	0.008	0.002	0.002

Standard errors in parentheses

*** p<0.01

** p<0.05

* p<0.1

Table 7 reports the main results restricted to the non-attritors. As expected, the number of observations is different from those in the whole sample. However, the main results (Table 7) are not substantially different from those found in the pure replication (Table 2). In general, the effect sizes and p-values are similar.

**Table 7:** Rates of new AIDS-defining illness or death at 48 weeks, according to CD4+ T-cell count restricted on non-attritors.

Variables	N	Earlier ART (%)	Later ART (%)	95% CI of diff	P-Value
All Patients	743	13.85	17.06	-2.00 to 8.42	0.22
<50 cells/mm^3^	270	16.29	27.4	1.26 to 20.96	0.02
≥50 cells/mm^3^	473	12.44	11.25	-7.04 to 4.65	0.68
Confirmed TB at study entry	348	12.92	18.23	-2.32 to 12.95	0.17
<50 cells/mm^3^	143	17.1	29.85	-1.15 to 26.64	0.07
≥50 cells/mm^3^	205	9.8	10.67	-7.51 to 9.26	0.83
Suspected TB at study entry	395	14.73	16.09	-5.81 to 8.53	0.70
<50 cells/mm^3^	127	15.25	0.25	-4.47 to 23.96	0.17
≥50 cells/mm^3^	268	14.5	11.67	-10.95 to 5.30	0.49
Low BMI (≤18.5) at study entry	306	16.26	0.25	-0.32 to 7.78	0.05
<50 cells/mm^3^	123	16.12	34.42	3.02 to 33.57	0.01
≥50 cells/mm^3^	183	16.34	17.72	-9.72 to 12.48	0.80

In short, we conclude that not accounting for loss to follow-up did not affect the original results. Therefore, as specified in the pre-analysis plan, no further corrections or adjustments were implemented. As in the original paper, the rest of analysis uses the full patient sample.

#### 3.2.2 Using an ANCOVA estimation, increasing power and correcting for baseline imbalance in male sex

Table 8 presents effect of earlier initiation of ART on new AIDS events using an ANCOVA estimation. Column 1 presents the effect of earlier ART initiation for all patients controlling for CD4 count at baseline. As in the pure replication, we find no effect of earlier ART initiation on the endpoint. As expected, a high CD4 count at baseline reduces the likelihood of a new AIDS event.

**Table 8:** Effect of earlier initiation of ART on new AIDS events using ANCOVA estimation.

	All patients	All patients by CD4 level	Patients with confirmed TB by CD4 level	Patients with suspected TB by CD4 level	Patients with low BMI (≤18.5) by CD4 level	All patients by CD4 level, controlling for sex
Treatment	-0.035	-0.004	-0.033	0.019	-0.043	-0.005
	(0.02)	(0.03)	(0.05)	(0.04)	(0.05)	(0.03)
CD4 <50 cells/mm^3^	0.123***	0.172***	0.206***	0.141***	0.171***	0.172***
	(0.03)	(0.04)	(0.05)	(0.05)	(0.07)	(0.04)
Trtment X CD4<50 cells/mm^3^		-0.096*	-0.077	-0.113	-0.099	-0.096
		(0.05)	(0.07)	(0.07)	(0.09)	(0.05)
Sex						0.015
						(0.03)
Constant	0.121***	0.105***	0.101***	0.109***	0.168***	0.097***
	(0.02)	(0.02)	(0.03)	(0.03)	(0.04)	(0.03)

Standard errors in parentheses

*** p<0.01

** p<0.05

* p<0.1

Note: The third fourth and fifth columns are based on TB or BMI status at baseline.

In column 2, the interaction (treatment × CD4 (<50 cells/mm^3^)) coefficient allows us to assess whether the impact of earlier ART initiation is different in the two CD4 groups. The interaction coefficient is statistically significant only at p=0.1. This suggests that earlier ART may reduce new AIDS event only for patients with a CD4 count of less than 50, but it’s not conclusive. When we consider only patients with confirmed TB at study entry (column 3) and patients with suspected TB at study entry (column 4), we find no differential effect of earlier ART initiation on new AIDS events by the level of CD4 count. These results are similar than those found in the pure replication and in the original paper. Unlike the original paper, when we estimate the effect of earlier ART on patients with low BMI (≤18.5) using regression, we also find no effect of earlier ART. Finally, in the pure replication, the earlier ART group has 7 percentage points more male study participants than the later ART group. However, column 6 shows that controlling for sex does not change the results.

#### 3.2.3 As treated analysis using instrumental variable

Of the 806 participants enrolled in the study, 783 initiated ART during the study. Of the 783 people who initiated ART, 17 initiated ART between 15 days and 55 days. These 17 patients initiated ART neither within 2 weeks after the initiation of treatment for tuberculosis nor later ART (between 8 and 12 weeks after the initiation of treatment for tuberculosis). Of the 17 patients, 11 were randomly assigned to the earlier ART group while the other six were assigned randomly to the later ART group. For the IV, we classified all 17 patients as not receiving earlier ART.

Table 9 presents the treatment on the treated estimates of the impact of earlier ART initiation on new AIDS events. Column 1 shows that there is no effect of earlier ART initiation when considering all patients. In column 2, I assess the heterogeneity by the level of CD4 count at baseline. The interaction coefficient is not statistically significant. This suggests that earlier ART initiation has no effect for patients with CD4 counts of less than 50 in the treatment on treated. I obtain the similar results for patients with confirmed TB at study entry (column 3), patients with suspected TB at study entry (column 4), and patients with low BMI (column 5).

**Table 9:** Treatment on treated estimates of the impact of earlier ART initiation on new AIDS events.

	All patients	All patients by CD4 level	Patients with confirmed TB by CD4 level	Patients with suspected TB by CD4 level	Patients with low BMI (≤18.5) by CD4 level
	(1)	(2)	(3)	(4)	(5)
Earlier ART initiation	-0.011	0.011	-0.019	0.034	-0.028
	(0.03)	(0.03)	(0.05)	(0.04)	(0.05)
CD4(<50 cells/mm^3^)		0.156***	0.176***	0.137***	0.156**
		(0.04)	(0.05)	(0.05)	(0.06)
CD4 (<50 cells/mm^3^)X Earlier ART Initiation		-0.079	-0.049	-0.107	-0.081
		(0.05)	(0.07)	(0.07)	(0.09)
Constant	0.137***	0.088***	0.087***	0.089***	0.146***
	(0.02)	(0.02)	(0.03)	(0.03)	(0.04)
Observations	783	783	364	419	321
R-squared	0.001	0.027	0.048	0.015	0.029

Standard errors in parentheses

*** p<0.01

** p<0.05

* p<0.1

#### 3.2.4 Heterogeneity of treatment effect for different windows of earlier ART initiation

**Fig 2:**
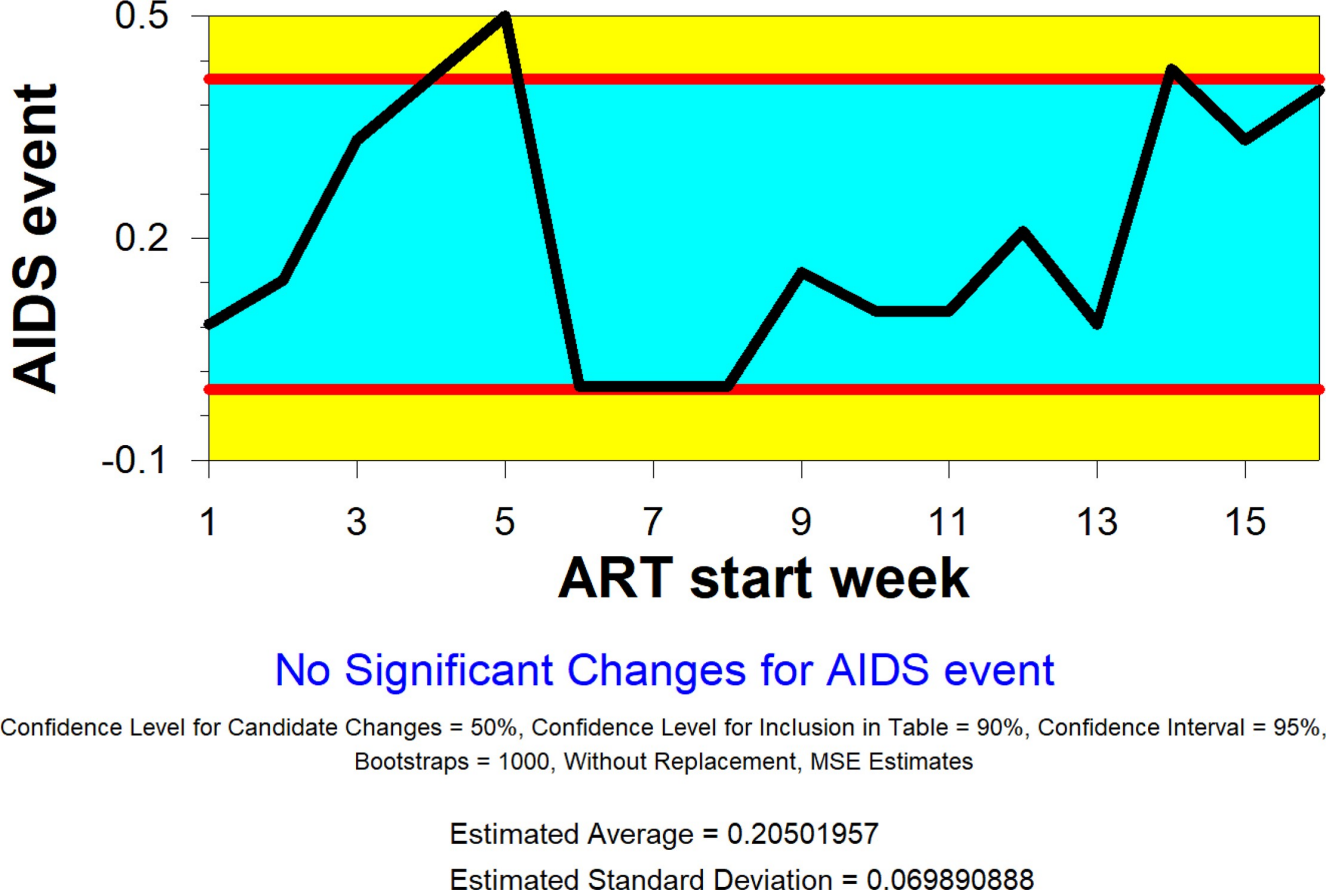
Plot of rate of AIDS events by ART start week.

The change point analysis assesses whether there is a specific point at which a change occurs. Fig 2 below presents the results. It shows the trend of the proportion of participants with an AIDS event by 48 weeks (endpoint), according to ART start week. The blue area represents a region expected to contain all the values assuming there is no critical change in the trend. The two red lines are called control limits. They represent the boundaries of the blue area. Points outside the control limits (the yellow region) indicate a change may have occurred. Associated with each change is a confidence level indicating the likelihood that a change occurred. Similar to statistics, 95% confidence means that there is only a 5% chance of a value falling outside the range even if no change took place. In addition, a 95% confidence interval is constructed that represents the time period within which a change occurred.

Here we group participants by ART start time in weekly increments from 1 to 16+ weeks. Weeks are measured as the time between TB treatment initiation and ART initiation. The 5 participants who started ART at 16 weeks or beyond are grouped together. 23 had an AIDS event before starting ART and were dropped from the analysis. Weeks 3-8 were largely empty, since the study called for starting ART within 2 weeks or after 8 weeks. Condensing these observation into one group did not change the result. Fig 2 displays the trend in proportions of participants with an AIDS event by endpoint by ART start week. The change-point analysis detects no systematic change. The trend in proportions of new AIDS events by ART start week may be influenced by baseline CD4 count, as suggested in the original paper. However, no changes are detected.

#### 3.2.5 Heterogeneity of treatment effect for different cut-off points of CD4 count

We use change point analysis to determine, in an endogenous manner, if there is a cut-off point from which earlier ART might have an impact on mortality. Prior assessments have selected the cut-off for earlier treatment somewhat arbitrarily. This analysis will contribute to filling the knowledge gap regarding the uncertainty around delaying ART for HIV-TB co-infected patients with CD4 counts between 50 and 220 pointed out in Uthman et al. [7], as well as indicate whether there is a true threshold at 50.

**Fig 3:**
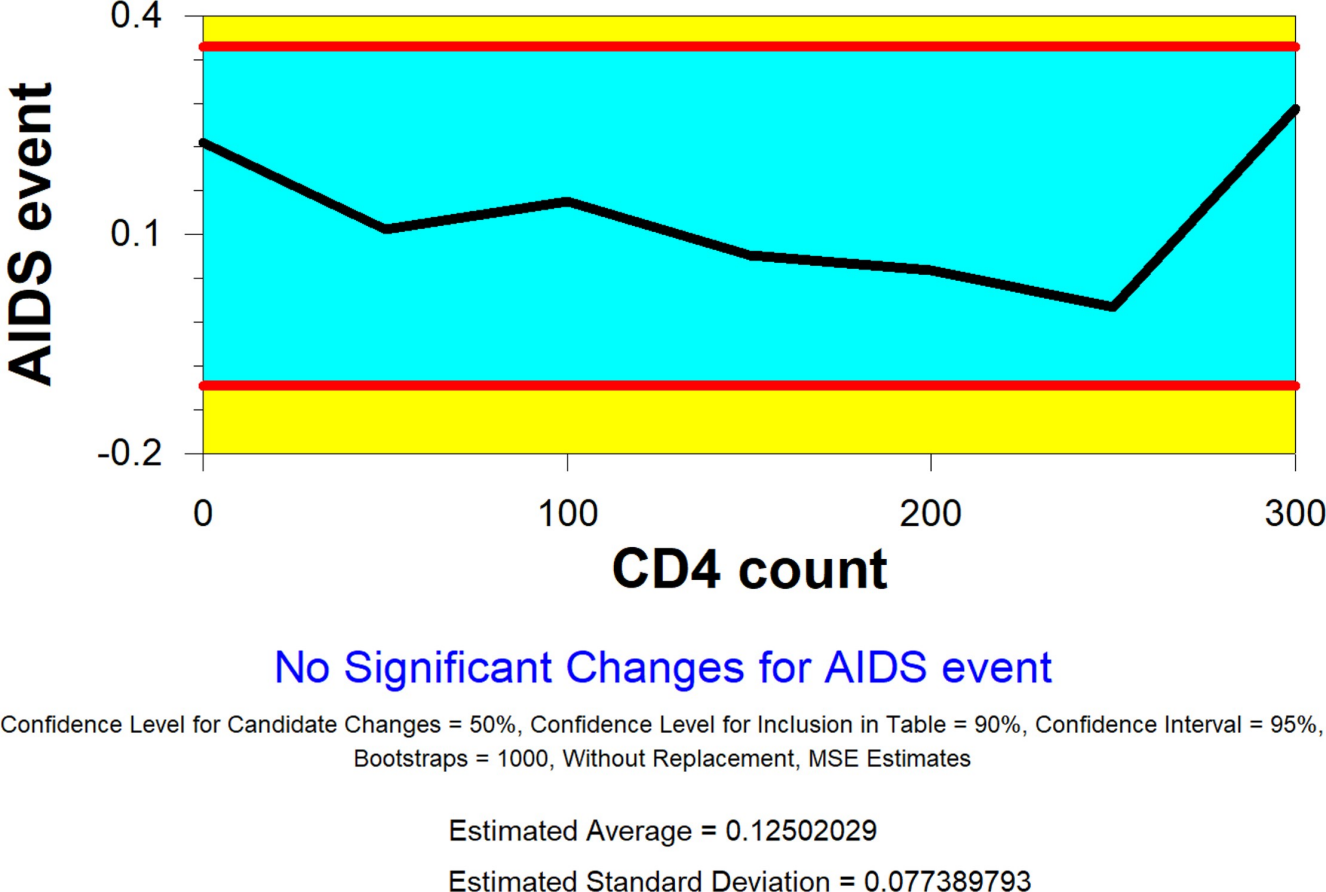
Plot of proportional AIDS events by CD4 count level.

Fig 3 depicts the trend of the rate of a new AIDS event by CD4 count at baseline.

We group participants by different cut-off points of CD4 count at the baseline in intervals of 50. Because there are very few patients with CD4 counts over 300, we decided to form one group with these patients. The change-point analysis detects no change.

**Fig 4:**
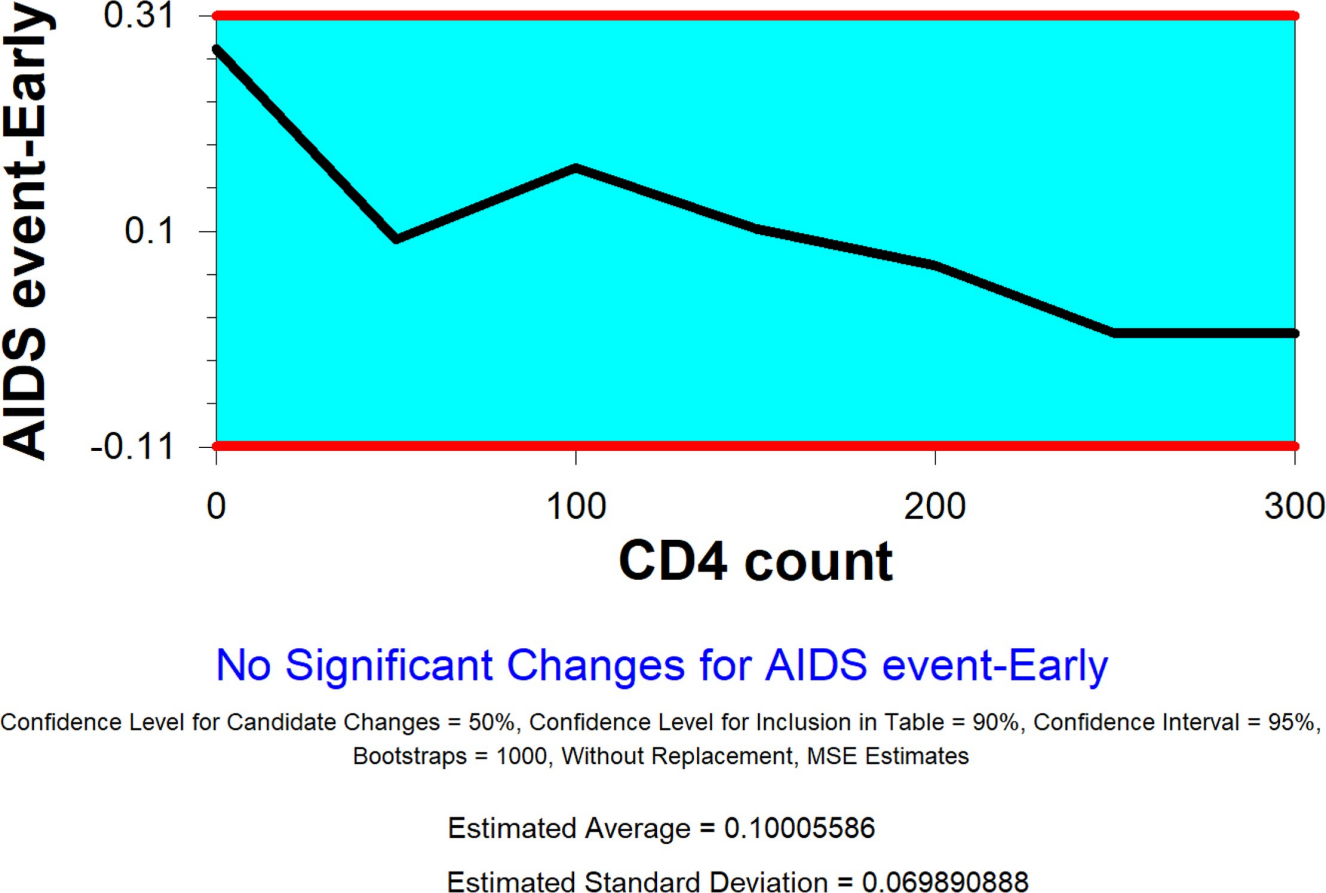
Plot of CD4 count level (treatment group).

We also conduct the change-point analysis by treatment group. Fig 4 displays the trend of the rate of a new AIDS event by CD4 count at baseline for the patients assigned in the treatment group. Again, no change is detected by the change-point analysis.

**Fig 5:**
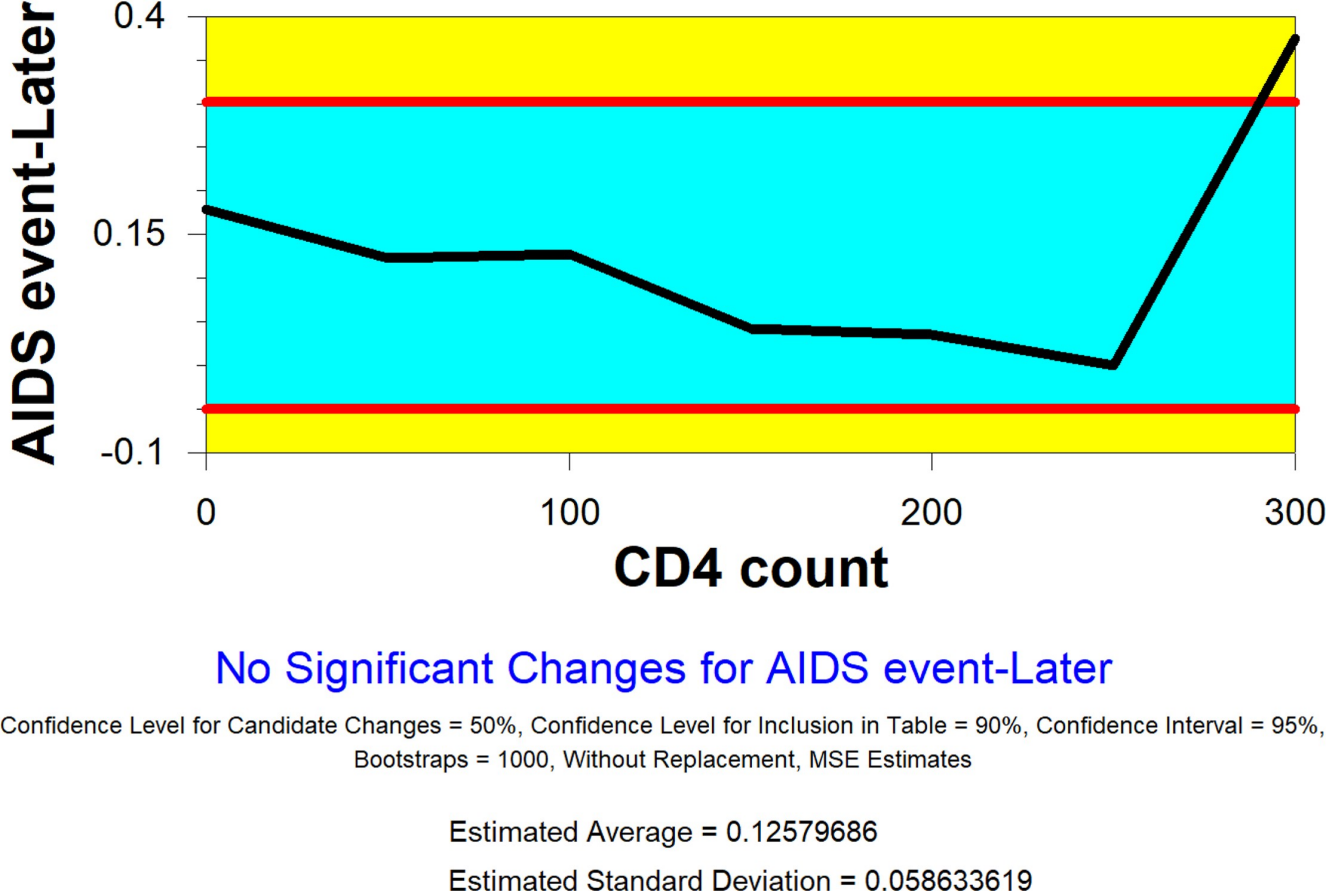
Plot of CD4 count (control group).

Fig 5 shows the trend of the rate of a new AIDS event by CD4 count at baseline for the patients assigned in the control group. The change-point analysis detects no systematic change of the rate of a new AIDS event by CD4 count at baseline.

## 4. Discussion

The results of this replication study have several important implications. The pure replication suggests that in general, the main results reported in the original paper do not suffer from any errors that might come from different sources, such as construction of variables, data cleaning and codes used to obtain findings. Thus, we are able to confirm that the data and methods described by Havlir et al. [5] are those used to produce the main findings reported in the original paper.

For the MEA, we used primarily econometric approaches to adjust for loss to follow-up in the analysis, increase power through an ANCOVA estimation, and estimate treatment on treated with an instrumental variables model. The results show that loss to follow-up does not affect the original results. There was low attrition overall, and no differential attrition between treatment and control groups.

We also found that using an ANCOVA estimation to increase the power of subgroup analyses produces effect sizes similar to those in the original paper. However, the level of significance for the main effect of earlier ART initiation for those with CD4 count < 50, is reduced to p =0.066 (significant at the 10% level, compared with the original p = 0.02, significant at 5%). This is in contrast to what is generally expected when the statistical power of a study is higher. The results of the as treated analysis using instrumental variables show that earlier ART initiation has no effect on the AIDS events for patients with a CD4 count of less than 50. Again, this is in contrast to what one might have expected. Because an as treated analysis measures the effect of an intervention on patients who actually received it, in general, it provides an estimate of the effect with a larger effect size than what is found in an intent to treat analysis.

However, the use of both an ANCOVA estimation and instrumental variables weakened the main results of the paper. These two results provide added evidence that the result presented in the original paper may not be robust to alternative methods of estimation.

Our more systematic exploration of different windows of earlier ART initiation and different cut-off points of CD4 count using change-point analysis showed that there is no strong association between the rate of a new AIDS event and different start times or different levels of CD4 count at baseline. Therefore, we do not have evidence that can lead us to recommend any particular start times or any different levels of CD4 count to use to guide when or how to prescribe earlier ART for HIV-TB co-infected patients. The choice of when and whether to start earlier ART initiation should be based primarily on factors such as potential drug interactions, overlapping potential for side-effects, high pill burden, and severity of illness, rather than thresholds and pre-set timeframes.

Thus, while we can replicate the original results, the results of this replication do not provide strong support that earlier ART initiation reduces the rate of new AIDS events, even for HIV positive TB patients with a CD4 count of less than 50. The result of this replication is aligned with more recent studies that show no evidence that earlier initiation of ART reduces mortality.

In light of these findings and the more recent literature, we suggest that WHO consider revisiting their recommendation to take into account these new findings. In addition, given the recent change in the landscape of HIV treatment, where every HIV patient is eligible for ART regardless of CD4 count, it is plausible that, over time, there will be a reduction of patients with very low CD4 counts. Therefore, the assessment of effectiveness of earlier initiation versus later initiation should give more weight to TB-IRIS and other criteria in determining the best approach to treat TB-HIV co-infected patients. We recommend that additional research based on criteria such as WHO staging and overall patient well-being be undertaken to assess the optimal timing for ART initiation. Finally, more studies are needed to assess the effect of initiation of treatment for TB in the current context of universal HIV treatment rather than the previous model of patients waiting until sick enough to qualify for treatment.

## Abbreviations

AIDS: Acquired immune deficiency syndrome
ANCOVA: Analysis of covariance
ART: Antiretroviral therapy
CD4 count: Cluster of differentiation 4, a count of a type of white blood cell
Grade 3: Severe adverse event
Grade 4: Life-threatening adverse event
HIV: Human immunodeficiency virus
IRIS: Immune reconstitution inflammatory syndrome
MEA: Measurement and estimation analysis
RNA: Ribonucleic acid
T cell: T lymphocyte—a type of white blood cell
TB: Tuberculosis
WHO: World Health Organization
